# The burden of breast, cervical, and colon and rectum cancer in the Balkan countries, 1990–2019 and forecast to 2030

**DOI:** 10.1186/s13690-023-01137-9

**Published:** 2023-08-24

**Authors:** Jovana Todorovic, Zeljka Stamenkovic, Aleksandar Stevanovic, Natasa Terzic, Katarzyna Kissimova-Skarbek, Fimka Tozija, Enkeleint A. Mechili, Brecht Devleesschauwer, Zorica Terzic-Supic, Milena Vasic, Vesna Bjegovic-Mikanovic, Milena Santric-Milicevic, the COST Action 18218 participants Burden of Disease Collaborator Network, Aaron Liew, Alexios Fotios Mentis, Alibek Mereke, Ana Catarina Sousa, Ana Laura Manda, Artemis Gkitakou, Balazs Adam, Bogdan Oancea, Bogdan Socea, Brigid Unim, Catalin Gabriel Smarandache, Che Henry Ngwa, Cornelia Melinda Adi Santoso, Daniel Sur, Dietrich Plass, Elena Pallari, Evangelia Nena, Federica Gazzelloni, Florian Fisher, Francesk Mulita, Giulia Collatuzzo, Grant Lewison, Grant Wyper, Hanen Samouda, Ionut Negoi, Jose Luis Penalvo, Juan Manuel Garcia Gonzalez, Juanita Haagsma, Klara Dokova, Lazslo Lorenzovici, Lorenzo Monasta, Miguel Reina Ortiz, Mihaela Hostiuc, Mika Gissler, Niko Speybroeck, Orsolya Varga, Peter Gaal, Polychronis Kostoulas, Ronan O’Caoimh, Sarah Cuscieri, Sonia Namorado, Tomislav Mestrovic, Vanessa Gorasso, Vesna Zadnik, Vildan Mevsim, Zubair Kabir

**Affiliations:** 1 Faculty of Medicine, Institute of Social Medicine, School of Public Health and Health Management, University of Belgrade, Dr Subotica 15, Belgrade, 11000 Serbia; 2 Institute of Public Health of Montenegro, Podgorica, Montenegro; 3 Jagiellonian University, Krakow, Poland; 4 Saints Cyril and Methodius University of Skopje, Skopje, North Macedonia; 5 University of Vlorë, Vlore, Albania; 6 Sciensano (Belgium), Brussels, Belgium; 7 Institute of Public Health of Serbia, Belgrade, Serbia; 8 Faculty of Medicine and Health Care, Al-⁠Farabi Kazakh National University, Almaty, Kazakhstan; 9 Institute for Health Metrics and Evaluation, University of Washington – GBD Collaborator, Seattle, USA; 10 UN ECOSOC - Economic and Social Council, New York, USA

## Abstract

**Background:**

Despite effective prevention and control strategies, in countries of the Balkan region, cancers are the second leading cause of mortality, closely following circulatory system diseases.

**Objective:**

To describe trends in the burden of breast, cervical, and colon and rectum cancer in the Balkan region and per country between 1990 and 2019, including a forecast to 2030.

**Methods:**

We described the 2019 Global Burden of Disease (GBD) estimates for breast, cervical, and colon and rectum cancers in eleven Balkan countries over the period 1990–2019, including incidence, years lived with disability (YLD), years of life lost (YLL), and disability-adjusted life years (DALYs) rates per 100,000 population and accompanied 95% uncertainty interval. With the Autoregressive Integrated Moving Average, we forecasted these rates per country up to 2030.

**Results:**

In the Balkan region, the highest incidence and DALYs rates in the study period were for colon and rectum, and breast cancers. Over the study period, the DALYs rates for breast cancer per 100,000 population were the highest in Serbia (reaching 670.84 in 2019) but the lowest in Albania (reaching 271.24 in 2019). In 2019, the highest incidence of breast cancer (85 /100,000) and highest YLD rate (64 /100,000) were observed in Greece. Romania had the highest incidence rates, YLD rates, DALY rates, and YLL rates of cervical cancer, with respective 20.59%, 23.39% 4.00%, and 3.47% increases for the 1990/2019 period, and the highest forecasted burden for cervical cancer in 2030. The highest incidence rates, YLD rates and DALY rates of colon and rectum cancers were continuously recorded in Croatia (an increase of 130.75%, 48.23%, and 63.28%, respectively), while the highest YLL rates were in Bulgaria (an increase of 63.85%). The YLL rates due to colon and rectum cancers are forecasted to progress by 2030 in all Balkan countries.

**Conclusion:**

As most of the DALYs burden for breast, cervical, and colon and rectum cancer is due to premature mortality, the numerous country-specific barriers to cancer early detection and quality and care continuum should be a public priority of multi-stakeholder collaboration in the Balkan region.

**Supplementary Information:**

The online version contains supplementary material available at 10.1186/s13690-023-01137-9.

**Table:** 

**Text box 1. Contributions to the literature**
• The first study to assess the burden of breast, cervical and cancer of colon and rectum in the Balkan region.
• The burden of breast, cervical and cancer of colon and rectum in the Balkan region is predominantly due to premature mortality.
• Forecasting shows increase in burden of breast, cervical and cancer of colon and rectum in some countries of the Balkan region.

## Introduction

The Sustainable Development Goal 3 explicitly aims to reduce mortality related to non-communicable diseases (NCDs), including cancers that represent a significant public health problem due to their influence on almost every developmental aspect of society [1]. The 2019 global estimates indicate colon and rectum cancer caused more than 24 million DALYs. Furthermore, each year, 20 million disability-adjusted life years (DALYs) are associated with breast cancer burden [2], mainly causing mortality in women, while cervical cancer was associated with 9 million DALYs [2]. However, cervical cancer incidence is the third worldwide [3], with diagnosed 570,000 new cases and approximately 311,000 deaths in 2018 among women globally [4]. According to death rates, colon and rectum cancer ranked fourth globally [5]. This is happening despite the well-known primary prevention strategies [6] and evidence-based programs for early diagnosis and screening for these cancers that can help countries ensure the onset of timely treatment and prevent the enormous losses of healthy lives and premature mortality [7, 8]. Considering the worrying burden of these cancers, performing a trend analysis could assist local and regional policymakers in planning or expanding programs for the early detection of cancers.

According to the World Health Organization, approximately 70% of cancer deaths happen in low- and middle-income countries [9]. In countries of the Balkan region, cancers are the second leading cause of mortality, closely following circulatory system diseases [10]. Balkan countries, versus the world population, seem to bear a disproportional cancer burden measured by the incidence and mortality data. For example, globally, due to colon and rectum cancer, there were around 1,148,515 new cases and 576,858 deaths in 2020 [11], while in Balkan countries only, which comprise about 0.3% of the world population, there were 56,960 new cases and 30,166 deaths of colon and rectum cancer in 2018, i.e., about 5% of estimated global mortality due to colon and rectum cancer there. As the most detrimental to their populations, Balkan countries established screening programs to diagnose and treat breast, cervical and colon and rectum cancers in the early stages [12]. However, even with screening, recent estimates show breast cancer ranked as the third leading cause of cancer mortality in Bulgaria, Croatia, Greece, Moldova, Montenegro, and Serbia, the fourth in North Macedonia, Romania, and Bosnia and Herzegovina, the fifth in Slovenia, and the eighth in Albania [13]. In contrast, there was a decrease in mortality due to cervical cancer from 1990 to 2017 in Balkan countries also belonging to East and Central Europe [14]. However, colon and rectum cancer affected more high-income Balkan countries than other countries in this region [15]. Contextually, the countries of this region share some geo-historical roots and, in the last decades, complex social, political, and economic transformations and a pronounced depopulation combined with ageing and emigration [16, 17]. Balkan countries include Albania, Bosnia and Herzegovina, Bulgaria, Croatia, Greece, Montenegro, Northern Macedonia, Romania, Slovenia, Serbia and the Republic of Moldova due to its strong historical connections with Romania [18]. The World Bank categorises countries in the Balkan region as high-income countries (Slovenia, Romania and Greece) and upper-middle-income countries (Albania, Croatia, Serbia, Bosnia and Herzegovina, Bulgaria, North Macedonia, Montenegro, and Moldova) [19]. In that regard, it would be relevant to outline the cancer burden situation, focusing on their contextual similarities and differences and the cancer burden forecast in the Balkan region if no change is implemented in the years between.

The aim of the study is to describe trends in the burden of breast, cervical, and colon and rectum cancer in the Balkan region and per country between 1990 and 2019, including a forecast by 2030.

## Methods

Using an ecological approach, we analysed the trends of incidence, years of life lost (YLL), years lived with disability (YLD), and disability-adjusted life years (DALYs) for breast, cervical, and colon and rectum cancer in eleven Balkan countries from 1990 to 2019, and their forecasts in 2030.

According to the Encyclopedia Britannica [18], the Balkan region includes Albania, Bosnia and Herzegovina, Bulgaria, Croatia, Greece, Montenegro, North Macedonia, the Republic of Moldova, Romania, Serbia and Slovenia. Balkan countries that are also Europe Union (EU) member states are Bulgaria (since 2007), Croatia (since 2013), Greece (since 1981), Romania (since 2007) and Slovenia (since 2004).

This study used the 2019 Global Burden of Disease (GBD) estimates updated by the Institute for Health Metrics and Evaluation (IHME). From the IHME databases (https://ghdx.healthdata.org/), we took estimated rates per 100,000 population and accompanied 95% uncertainty interval of the following metrics: incidence, DALYs, YLD and YLL for breast cancer (International Classification of Disease, 10-revision, i.e., ICD-10 C50-C50.9, D05-D05.9, D24-D24.9, D48.6 and D49.3), cervical cancer (ICD-10 C53, C53.0, C53.1, C53.3, C53.4, C53.8, C53.9, D06, D06.0, D06.1, D06.7, D06.9, D26.0), and colon and rectum cancer (ICD-10 C18-C21, D01, D12, D37.3-D37.5, Z12.1-Z12.13, Z85.03-Z85.048, Z86.010) [20, 21]. DALYs is a composite measure of the population burden of disease, it is the sum of two components, years of life lost due to premature mortality and years lived with disability due to healthy life lost, each estimated using the GBD methods presented elsewhere [20]. Briefly, the YLL is the sum of the number of deaths at each age due to selected cancer causes multiplied by the remaining years from the standard life expectancy (the GBD life tables) at the age of death. YLD is the product of the prevalence number multiplied by the disability weights for the given disease, also taken from the GBD Study [20].

Time series trends were analysed using the traditional forecasting model - the Autoregressive Integrated Moving Average - ARIMA (expert modeller), and the end year specified was 2030. The input into the ARIMA forecasting model were the YLL, YLD and DALYs rates in the period from 1990 to 2019, taken form the 2019 GBD Study. The first case after the end of estimation period of 40 years (1990–2030), i.e., the predicted value with accompanied 95% confidence intervals was presented. The analyses were done using the Statistical Package for Social Sciences 22.0.

## Results

This section displays the burden of selected cancer groups by country, estimated highest and lowest incidence, YLD, YLL, and DALYs rates, including the per cent changes of the burden for the 30 years from 1990 to 2019, then burden estimates for the latest year available, 2019, and finally, their forecasts by 2030.

### The burden of breast cancer in Balkan countries

**Fig. 1:**
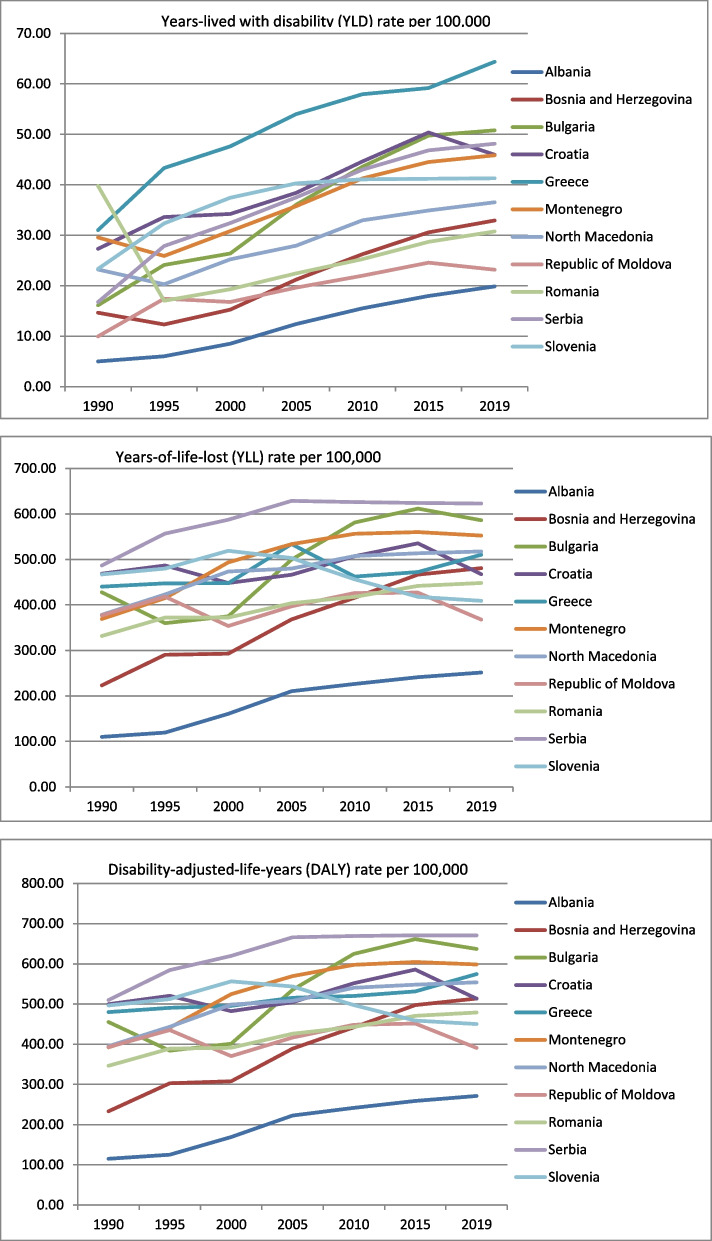
The trends in years lived with disability, years of life lost, and disability-adjusted life years due to breast cancer in Balkan countries in the period 1990–2019 (YLD, YLL and DALYs rates per 100,000 population with corresponding 95% uncertainty interval)

Breast cancer incidence and YLD rates increased in all Balkan countries throughout the observed period (Supplemental files 1 and 2; Fig. 1). In the period 1990–2019, the DALYs and YLD rates per 100,000 population were among the highest in Serbia (the overall increases were 31.57% and 106.26%, respectively) but the lowest in Albania despite significant increases of 135.98% and 297.20%, respectively (Fig. 1). While the highest YLL rates were also continuously recorded in Serbia (Fig. 1), with a 27.98% increase over the 1990–2019 period, the lowest was also in Albania despite the 128.65% increase for 1990–2019 (Supplemental files 2–4). Only three countries showed an overall decrease in YLL rates - Croatia, Moldova, and Slovenia, and two of them (Moldova and Slovenia) had also decreased overall DALYs rates (Supplemental files 3 and 4). Slovenia was the best performer, with an overall decrease of -9.33% (450.19 DALYs per 100,000 in 2019) and -12.44% (408.89 YLL per 100,000 in 2019).

In 2019, the highest incidence of breast cancer (85.00 per 100,000) and the highest YLD rate (64.00 per 100,000) were observed in Greece. Serbia has the highest DALYs rate (671.00 per 100,000) and the highest YLL rates (623.00 per 100,000) (Table 1).

**Table 1:** The burden of breast cancer in Balkan countries in 2019 (rates / 100,000 population and 95% uncertainty interval) and forecasts in 2030 (rates / 100,000 population and 95% confidence interval)

Country	Incidence	DALY 2019	Forecasted DALY 2030	YLL 2019	Forecasted YLL 2030	YLD 2019	Forecasted YLD 2030
Albania	28.10 (20.41–37.90)	271.24 (199.18–366.32)	334.53 (200.41–408.32)	251.38 (185.33–340.23)	316.01 (273.26–358.75)	19.86 (12.28–29.88)	24.78 (19.11–29.65)
Bosnia and Herzegovina	49.54 (37.97–63.78)	513.67 (396.77–659.09)	622.74 (558.01–687.46)	480.74 (372.77–609.93)	585.86 (542.66–629.06)	32.93 (21.92–46.96)	41.55 (32.90–50.20)
Bulgaria	73.86 (57.73–93.47)	636.85 (499.68–806.77)	590.20 (150.35–1030.06)	586.06 (459.01–739.73)	536.39 (117.80–954.99)	50.80 (33.31–72.54)	53.53 (32.42–74.65)
Croatia	67.44 (52.12–85.83)	513.29 (398.19–648.19)	513.29 (394.79–631.80)	467.31 (358.65–589.86)	467.34 (355.21–579.46)	45.98 (30.35–64.60)	53.17 (42.27–66.09)
Greece	**85.13 (65.97–108.93)**	574.51 (525.37–631.90)	610.40 (568.39–652.40)	510.13 (468.72–548.63)	536.69 (497.06–576.32)	**64.38 (43.24–92.61)**	**77.39 (70.51–84.78)**
Montenegro	66.58 (53.05–81.96)	598.28 (477.93–734.52)	577.33 (341.30–813.36)	552.44 (440.74–679.92)	528.17 (297.09–759.25)	45.85 (30.63–64.51)	49.16 (42.72–55.60)
North Macedonia	54.24 (40.96–70.27)	553.96 (419.65–710.83)	544.23 (480.13–608.32)	517.42 (393.05–661.17)	510.56 (437.41–583.70)	36.54 (23.36–53.99)	44.49 (41.98–47.00)
Republic of Moldova	33.74 (28.52–39.82)	390.65 (333.50–462.97)	390.65 (255.54–525.76)	367.49 (312.86–434.87)	384.09 (336.55–431.63)	23.16 (15.59–32.34)	24.63 (3.92–45.35)
Romania	45.49 (36.85–55.28)	478.90 (387.45–585.89)	529.19 (473.26–585.12)	448.13 (363.03–545.98)	492.31 (437.55–547.07)	30.76 (20.87–42.97)	37.05 (35.56–38.55)
Serbia	71.49 (40.96–70.27)	**670.84 (530.31–847.04)**	**731.89 (622.84–840.95)**	**622.70 (492.48–788.14)**	**674.34 (567.15–781.53)**	48.14 (31.82–69.39)	59.23 (54.42–64.04)
Slovenia	59.43 (45.64–78.99)	450.19 (348.30–589.97)	450.11 (353.14–547.08)	408.89 (314.89–541.23)	409.87 (307.44–512.31)	41.30 (26.52–59.76)	43.37 (32.63–54.11)

By 2030, the DALYs rates for breast cancer are forecasted to decrease in Bulgaria, Montenegro, and North Macedonia by 7.35%, 3.50%, and 1.76%, respectively (590.20 per 100,000, 577.33 per 100,000, and 544.23 per 100,000, respectively), along the forecasted decrease of YLL rates for 8.48% in Bulgaria (536.39 per 100,000), for 4.39% Montenegro (528.17 per 100,000), and 1.33% in North Macedonia (510.56 per 100,000).

However, the YLD rates for breast cancer are forecasted to increase in all Balkan countries by 2030, among which Greece had the highest forecasted 77.39 YLD per 100,000 (Table 1). The worst performer among Balkan countries in 2030 will be Serbia concerning the highest forecasted DALYs rate (731.89 per 100,000) and the highest YLL rate (674.34 per 100,000) (Table 1).

### The burden of cervical cancer in Balkan countries

**Fig. 2:**
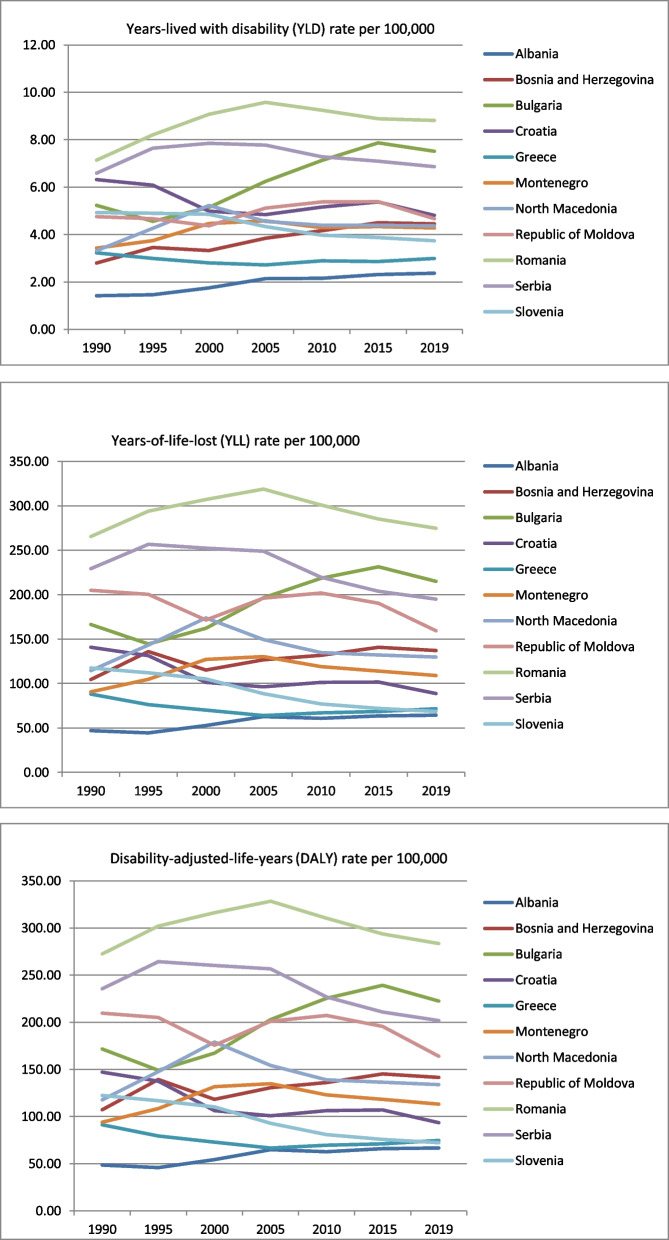
The trends in years lived with disability, years of life lost, and disability-adjusted life years due to cervical cancer in Balkan countries in the period 1990–2019 (YLD, YLL and DALYs rates per 100,000 population with corresponding 95% uncertainty interval)

Throughout the observed period, the cervical cancer incidence rates, YLD rates, YLL rates, and DALYs rates were decreasing in four Balkan countries – Croatia, Greece, Moldova, and Slovenia (Supplemental files 5–8), where Slovenia had the highest decrease in DALYs rates, -40.93% (to 72.30 DALYs per 100,000 in 2019) and YLL rates, -41.64% (to 68.56 YLL per 100,000). Serbia has also shown overall decreases in YLL and DALYs rates, but not in YLD rates. Given the per cent change for the 1990–2019 period, the highest incidence rates, YLD rates, YLL rates, and DALYs rates of cervical cancer were in Romania, with respective 20.59%, 23.39%, 4.00%, and 3.47% increases (Fig. 2) (Supplemental files 5–8).

In the observed period, the lowest DALYs and YLL rates due to cervical cancer were in Albania, Slovenia, and Greece (Supplemental files 7 and 8). Still, while Albania has reported overall increases in these rates (for 37.85% in DALYs rates and 36.99% in YLL rates), Slovenia and Greece reported overall decreases for 1990–2019, i.e., -40.93% in DALYs rates and -41.64% in YLL rates, and -18.15% in DALYs rates, and -19.55% in YLL rates, respectively.

In 2019, Romania had the highest incidence rate (20.44 per 100,000), the highest DALYs rate (283.47 per 100,000), the YLL rate (274.66 per 100,000) and YLD rate (8.81 per 100,000) (Table 2). In the same year, Albania had the lowest incidence of cervical cancer (5.16 per 100,000) among the Balkan countries. Albania also had the lowest DALYs rate (66.69 per 100,000), YLL rate (64.33 per 100,000) and YLD rate (2.37 per 100,000).

**Table 2:** The burden of cervical cancer in Balkan countries in 2019 (rates / 100,000 population and 95% uncertainty interval) and forecasts in 2030 (rates / 100,000 population and 95% confidence interval)

Country	Incidence	DALY 2019	Forecasted DALY 2030	YLL 2019	Forecasted YLL 2030	YLD 2019	Forecasted YLD 2030
Albania	5.16 (3.57–7.14)	66.69 (45.75–92.41)	75.83 (59.43–95.48)	64.33 (44.18–89.48)	72.98 (57.02–92.14)	2.37 (1.43–3.62)	2.89 (2.27–3.62)
Bosnia and Herzegovina	10.19 (6.84–13.31)	141.40 (96.52–185.10)	178.02 (53.71–302.34)	136.95 (93.28–178.98)	152.95 (117.54–195.95)	4.45 (2.71–6.69)	5.33 (4.27–6.58)
Bulgaria	16.78 (11.20–21.99)	222.57 (152.24–291.16)	184.88 (46.82–322.93)	215.06 (147.33–282.68)	178.11 (44.35–311.86)	7.51 (4.17–11.26)	6.80 (2.57–11.03)
Croatia	10.01 (7.47–13.13)	93.53 (70.52–121.44)	73.21 (45.25–101.17)	88.72 (66.51–114.79)	68.97 (42.23–95.72)	4.81 (3.03–7.26)	4.24 (3.01–5.46)
Greece	6.67 (5.11–8.72)	74.72 (67.39–84.12)	80.61 (43.61–117.61)	71.73 (64.79–80.85)	77.49 (42.09–112.89)	2.99 (1.90–4.43)	2.99 (2.51–3.47)
Montenegro	9.13 (7.25–11.62)	113.31 (89.74–141.76)	101.77 (34.34–169.21)	109.04 (86.53–136.43)	97.55 (32.07–163.03)	4.28 (2.84–6.26)	4.21 (1.98–6.44)
North Macedonia	9.90 (7.08–13.30)	134.06 (96.44–179.050	130.64 (11.17–250.11)	129.70 (93.14–173.08)	126.21 (10.38–242.04)	4.36 (2.69–6.53)	4.24 (0.63–8.22)
Republic of Moldova	10.81 (8.55–13.36)	164.02 (132.20–201.63)	164.02 (105.94–222.10)	159.35 (128.88–195.62)	159.35 (102.58–216.12)	4.67 (2.93–6.77)	4.67 (3.32–6.20)
Romania	**20.44 (14.22–25.81)**	**283.47 (195.57–360.22)**	**254.89 (115.57–394.20)**	**274.66 (189.36–347.23)**	**246.27 (111.40–381.15)**	**8.81 (5.37–12.85)**	**8.62 (4.10–13.14)**
Serbia	15.67 (11.41–20.55)	201.86 (146.57–264.07)	201.86 (143.20–260.52)	194.99 (142.19–254.76)	194.99 (137.97–252.02)	6.87 (4.24–10.20)	6.87 (5.28–8.46)
Slovenia	7.63 (5.52–10.63)	72.30 (53.06–99.05)	53.30 (36.63–69.97)	68.56 (50.65–94.11)	50.01 (34.10–65.92)	3.74 (2.31–5.73)	3.29 (2.51–4.08)

Romania has the highest forecasted burden for cervical cancer in 2030 among the Balkan countries (254.89 DALYs per 100,000, 246.27 YLL per 100,000, and 8.62 YLD per 100,000). The burden of cervical cancer is forecasted to decrease by 2030 in Bulgaria, Croatia, Montenegro, North Macedonia, and Slovenia (Table 2).

### The burden of colon and rectum cancer in Balkan countries

Throughout the observed period, the colon and rectum cancer incidence rates, YLD rates, YLL rates, and DALYs rates were increasing in all Balkan countries (Supplemental files 9–12).

**Fig. 3:**
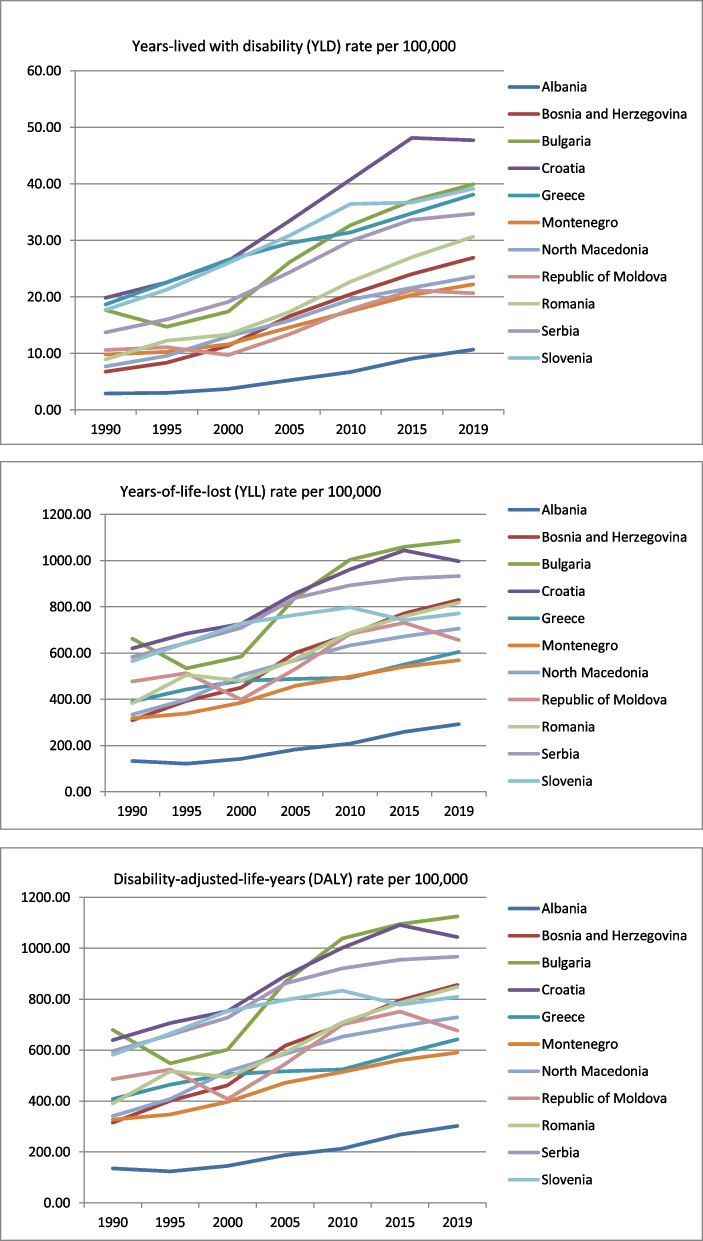
The trends in trends in years lived with disability, years of life lost, and disability-adjusted life years due to colon and rectum cancer in Balkan countries in the period 1990–2019 (YLD, YLL and DALYs rates per 100,000 population with corresponding 95% uncertainty interval)

The highest incidence rates, YLD rates and DALYs rates of colon and rectum cancers were almost continuously recorded in Croatia (an increase of 130.75% in incidence rates, 48.23% in YLD rates, and 63.28% in DALYs rates) (Fig. 3). In Bulgaria, an increase of 110.55% in incidence rates and 65.48% in DALYs rates were recorded, and in Slovenia, an increase of 104.17% in incidence rates and 39.26% in DALYs rates. However, the lowest rates were estimated in Albania despite a rise of 249.17% in incidence rates and 123.54% in DALYs rates (Fig. 3) (Supplemental files 9–12). The highest YLL rates of colon and rectum cancer were in Bulgaria, followed by Croatia and Serbia, with increases of 63.86%, 60.81%, and 12.62%, respectively over the 1990–2019 period (Fig. 3) (Supplemental files 9 and 12).

In 2019, the highest incidence of colon and rectum cancer among Balkan countries was in Croatia (103.79 per 100,000), as was the highest YLD rate (47.72 per 100,000) (Table 3). The highest DALY and YLL rates of colon and rectum cancer among Balkan countries in 2019 were in Bulgaria (1125.41 per 100,000 and 1085.49 per 100,000, respectively).

**Table 3:** The burden of colon and rectum cancer in Balkan countries in 2019 (rates / 100,000 population and 95% uncertainty interval) and forecasts in 2030 (rates / 100,000 population and 95% confidence interval)

Country	Incidence	DALY 2019	Forecasted DALY 2030	YLL 2019	Forecasted YLL 2030	YLD 2019	Forecasted YLD 2030
Albania	23.15 (17.29–30.43)	302.34 (225.05–397.30)	394.12 (233.64–554.60)	291.68 (217.41–383.50)	379.18 (221.36–536.99)	10.67 (6.86–15.53)	17.37 (8.62–31.65)
Bosnia and Herzegovina	62.35 (49.06–77.90)	856.03 (673.97–1072.27)	1069.95 (979.25–1160.64)	829.10 (653.26–1040.25)	1034.71 (945.54–1123.89)	26.93 (18.49–37.77)	36.30 (29.48–43.13)
Bulgaria	89.39 (71.75–110.18)	**1125.41 (897.78–1393.01)**	1211.10 (465.16–1957.04)	**1085.49 (866.39–1341.75)**	**1163.28 (433.98–1892.58)**	39.93 (27.35–56.18)	41.08 (17.68–64.48)
Croatia	**103.79 (82.47–128.83)**	1044.32 (828.89–1305.46)	**1246.26 (1044.94–1447**.57)	996.60 (789.01–1244.96)	1139.55 (952.97–1326.12)	**47.72 (32.97–47.72)**	**51.59 (33.76–69.43**)
Greece	76.45 (60.72–96.16)	642.32 (589.93–689.17)	731.21 (679.84–782.58)	604.21 (559.92–643.23)	685.73 (636.26–735.20)	38.10 (26.34–52.79)	44.95 (42.73–47.17)
Montenegro	48.27 (39.50–58.35)	590.54 (485.85–707.08)	652.98 (443.95–862.01)	568.33 (467.44–643.23)	625.96 (421.32–830.61)	22.22 (15.27–29.98)	27.00 (22.10–31.89)
North Macedonia	52.71 (41.73–65.78)	729.09 (572.89–916.37)	883.74 (817.92–949.57)	705.53 (555.57–886.31)	853.73 (789.09–918.37)	23.57 (16.02–33.11)	28.75 (20.39–37.10)
Republic of Moldova	45.85 (39.91–52.25)	676.68 (588.06–770.54)	748.81 (519.15–978.47)	656.02 (570.78–746.72)	656.02 (430.33–881.72)	20.65 (14.85–27.38)	21.04 (4.51–37.57)
Romania	67.78 (55.86–80.79)	848.20 (695.47–1022.47)	1145.41 (901.20–1437.02)	817.55 (669.65–987.32)	1098.46 (861.91–1381.49)	30.65 (21.78–41.79)	43.09 (36.58–43.09)
Serbia	77.62 (62.33–96.56)	967.17 (771.59–1204.91)	1164.76 (1007.46–1340.10)	932.46 (742.73–1160.30)	1117.38 (965.09–1287.32))	34.70 (23.56–48.14)	41.07 (29.01–53.14)
Slovenia	83.22 (64.91–107.10)	810.36 (630.96–1047.96)	897.03 (755.02–1039.03)	771.21 (598.65–994.53)	849.73 (710.90- 988.57)	39.16 (25.94–55.77)	47.85 (33.19–62.50)

The burden of colon and rectum cancer will increase in all Balkan countries by 2030. The forecast analysis of colon and rectum cancer in 2030 showed the highest DALY rates and YLD rates in Croatia (1246.26 DALYs per 100, 000 and 51.59 YLD per 100,000) and YLL rates in Bulgaria (1163.28 per 100,000) (Table 3).

## Discussion

Among the main findings of the study, the first one is that according to the rates of incidence and DALYs in the overall period and 2019, the highest-burden has been caused by colon and rectum cancers and breast cancer compared to cervical cancer, emphasising the need for improving the health promotion and access to prevention and treatment of these conditions. Furthermore, premature mortality due to the burden of cancers in the Balkan region, i.e., the YLL rates, mainly contributed to the DALYs rates. Observed YLL rates had an increasing trend from the beginning of the studied period, particularly in countries of former Yugoslavia, such as Bosnia and Herzegovina, Croatia, and Serbia, which also suffered from army conflicts during the 1990s. Also, an important finding is that eleven Balkan countries had diverse directions and paces of change in trends of YLL rates over the observed period, where the decline in forecasts by 2030 was estimated for cervical cancers only in six countries (Bulgaria, Croatia, Montenegro, North Macedonia, Romania, Slovenia), and for breast cancer only in three countries (Bulgaria, Montenegro, North Macedonia), while YLL rates for colon and rectum cancer are not forecasted to decline by 2030 in any Balkan country. A high burden due to colon and rectum cancers in the Balkan region between 1990 and 2019 is predicted to increase further in all countries by 2030. In addition, the 1990–2019 per cent change in incidence rates exceeded the per cent change in YLD rates due to breast cancer in eight countries, due to cervical cancer in two countries, and due to colon and rectum cancer in one country. Regarding these findings, further research might help understand whether national screening programmes contributed to the incidence rise and timely treatment of people with cancers. Another result is that the five EU countries in the Balkan region also do not follow the same pace of change or direction of trends regarding the burden of studied cancers, and it indicates the need for a more collaborative exchange of best cancer prevention and treatment practices in the Balkan region. In addition, while the trends in cervical cancer burden are declining in EU countries in the Balkan region, the burden of breast, colon and rectum, and cervical cancers remains a significant public health problem for remaining Balkan countries that are also among the countries in Europe with the lowest screening coverage [22].

The study findings align with the previous estimations describing the cancer trends in countries of the Balkan region up to 2019. For example, from the 2017 GBD systematic analyses [13, 23], we knew that DALYs associated with colon and rectum cancer in the Balkan countries varied from 1860 DALYs in Montenegro in 1990 to 135,000 DALYs in Romania in 2017. Our study results, based on the 2019 GBD Study estimations that derive from the improved methodology for the GBD [2], confirm that DALYs rates due to breast cancer were the highest in Serbia and due to colon and rectum cancer the highest in Bulgaria, that is there was no improvement over the 2 years, 2017–2019, in these two countries. The added value of our study is that it further systematically portrays per cent change in the 5-year trends of incidence, YLL and YLD, and DALYs rates per country and for each cancer group, for which the effective screening and treatment strategies are well-known. For example, our study confirmed previous findings [2] that the lowest DALY rates associated with breast cancer and with colon and rectum were in Albania, and add to it that these rates increased over the 30 years, between 1990 and 2019, at 135.98% and 123.54%, respectively and that will continue to grow by 2030 unless the cancer prevention and control strategies are better implemented. Though we know that total DALYs due to cervical cancer will decrease globally [23], our findings showed that a decrease will continue until 2030 in Serbia, Romania, Bulgaria, Croatia, Slovenia and Greece, while an increase by 2030 is forecasted only in Albania, Bosnia and Herzegovina, and Greece.

The study results show the highest percentage increases in the incidence rates due to all observed cancers from 1990 until 2019 in almost all observed countries, making it a Balkan-wide public health problem for many reasons. During the 1990s, there was a breakup of Yugoslavia marked by social turmoil and civic army conflicts in many of the countries belonging to the former Yugoslavia, and overstrained health and care delivery accompanied by lowering investments in the development of these sectors. Our study estimates are consistent with those in previous studies. For example, in 2018, Serbia was emphasized as the country with the continual annual increase of the standardized incidence rate of breast cancer [13], due to which it was the leading cause of cancer mortality in the women population [12].

Our study also showed that the worst country performers of the Balkan region in 2019 were Serbia due to the highest burden of breast cancer, Romania due to the highest burden of cervical cancer, and Croatia due to the highest burden of colon and rectum cancer. Furthermore, the study shows that an increase in the DALYs rates of breast cancer is expected in five of the analysed twelve Balkan countries (that is, Albania, Bosnia and Herzegovina, Greece, Romania, and Serbia) mainly due to increases of YLL rates. At the same time, forecasts for the remaining Balkan countries, i.e., Bulgaria, Croatia, Montenegro, North Macedonia, Republic of Moldova, and Slovenia, indicate the increase of YLD rates suggesting country efforts will be resulting in saving lives of their population but expanding loss of healthy life years. These discrepancies in forecasted increases among countries might be explained by the fact that population-based organized programs for breast cancer screening are not established in every country. For example, Bosnia and Herzegovina and Greece currently do not have any population-based organized screening [24]. In Albania, the screening is just recommended since 2013 [25, 26] while Romania has piloted the screening program in 2016 [24]. Although Serbia has had population-based organized screening programs since 2012 [27], various barriers to its use [28] including low adequate authorised by the National Insurance Fund [29, 30], might explain the third highest incidence rates of breast cancer recorded in 2019 in Serbia, behind the EU member states, Greece and Bulgaria. The highest DALYs rates in Serbia, with the dominant share of YLL rates, indicate insufficient progress in effectively preventing breast cancer. Another probable reason for the increase is the late stage of breast cancer diagnosis, given that just every 6^th^ woman of the target population had organised mammography in Serbia [27, 31]. These study findings for Serbia, as well as other countries with similar patterns, suggest the necessity of an expert assessment of the causes of ineffective screening and treatment programs in the country, revision of guidelines and legal instruments for the successful implementation of improved services provision for early detection and prevention of breast cancer for the benefit of the entire population.

Our study indicates the varied burden of cervical cancer in the region over the three decades. Its progress has been successfully halted in some Balkan countries, which means an opportunity for cross-regional collaboration and learning about best practices, essential for Romania, Serbia, Bulgaria, and Moldova, which had the worst estimates of DALYs, YLL, and YLD rates in 2019. Slovenia is an excellent example among Balkan countries, with the lowest forecasted burden. It was the first country in the region to introduce a population-based organised screening program in 2003 [17]. In that regard, the forecasted increased burden of cervical cancer in Greece and Bosnia and Herzegovina might be due to the lack of established population-based organised screening programs [16]. In 2013, Albania introduced the Recommendations for Implementing Breast and Cervical Screening Programs [17] and, until 2018, has established opportunistic cervical screening [26]. Recently, Albania introduced Human Papilloma Virus (HPV) screening at the primary healthcare level, based on the collaboration among gynaecologists, public health professionals, and other primary healthcare physicians. The recent implementation and the heterogeneity of HPV vaccination coverage programs [32] might help explain the overall increase of YLD rates in Albania by 2019 and 2030. In addition, in many countries, HPV vaccination might not be mandatory, or it is paid for if included.

Our forecasts analysis showed increased colon and rectum cancer burden in all examined countries. Bulgaria had the highest colon and rectal cancer burden in 2019, probably due to the low consumption of the average amount of fruits and vegetables per person per year, the lowest compared to other Balkan countries [10]. In addition, Bulgaria was also the second leading country among the Balkan countries in the prevalence of obesity [10], right behind Greece [10]. However, Greece is among the countries with the third lowest colon and rectal cancer burden in the Balkans, like Albania and Montenegro. In Albania, Montenegro, and Greece, the available fruits and vegetables are the highest per person per year [10]. According to forecasts, the burden of colon and rectal cancer will also increase in these countries unless adequate primary and secondary preventive measures are not implemented. Albania is projected to have the lowest burden of colon and rectal cancer in 2030, but the percentage change in DALYs rates is projected to be almost a quarter higher. Promoting healthy lifestyles, including a diet rich in fruits and vegetables and maintaining average body weight, may reduce the burden of colon and rectal cancer [5]. As our analysis shows that the burden of breast, cervical and colon and rectum cancers will remain significant and/ or increase in Balkan countries, and as these cancers are all associated with multiple preventable factors, the analysis urges for the establishment and implementation of the preventive measures, regarding the diet, physical activity, and adequate and implemented screening procedures [33].

The main findings show differences in overall increases in rates of DALYs and YLD suggest a need for international collaborative action for the exchange of regional and country-specific best practices toward the removal of individual, organisational, and systemic barriers to cancer early detection and quality treatment and to provide cost-effective cancer care continuum [28]. The best prevention and control strategies and practices help raise awareness and decrease barriers to early cancer detection in different contexts and trend patterns [5, 33–35]. Health education, early diagnosis, and timely and appropriate treatment help reduce mortality due to breast, colon and rectum, and cervical cancer [1].

Awareness of the risk factors, protective lifestyle practices, advances in the understanding of cancer disease pathogenesis, repeated testing at frequent intervals with adequately high coverage, quality-control procedures, and the availability of conventional and innovative cancer therapy have been observed earlier as factors enabling screening coverage [36–40], as opposed to poor healthcare infrastructure, absence of necessary logistics for transport, storage, and administration of tests, drugs or vaccines, low accessibility to services and lack of workforce capacities, competencies and facilities for cancer screening and treatment in remote areas [12, 41]. Treating cancer diseases often accompany significant out-of-pocket spending of patients and their families, either for supplements or visits to private practice, creating equality in patient treatment opportunities. For example, in Serbia, the list of medicinal products available via national health insurance includes immunological and bioengineered drugs, gene therapy, and blood and blood products [42, 43]. At the same time, some medications are provided only for specific indications and in particular institutions, e.g. monoclonal antibody bevacizumab is indicated for metastatic cancer of the colon and rectum along with fluoropyrimidine chemo-therapy or certain ovarian cancers and only in authorised institutions [42]. Enabling the local manufacturing of tests, drugs, and vaccines will likely encourage the uptake of national cancer control programs [44]. Also, combining interventions into a single program positively influences screening uptake in the targeted age group. Over the past decade, evidence rapidly emerges about the effectiveness of multiple invasive, semi- and non-invasive screening modalities, including the removal of precursor lesions as cancer causes, the potential of vaccines and the role of artificial intelligence and new technologies with better specificity in the detection of neoplasms, new screening algorithms [14] and tools (e.g. HPV testing for cervical cancer screening [17]), as well as complimentary engagement of trained of paramedical workers where needed to help reduction in cancer incidence and mortality [45].

The strength of our study includes the Balkan-wide comprehensive assessment of the burden of cancers for which effective prevention and control strategies are available. The study results provide evidence for critical national and international authorities regarding cancer control and prevention programs by describing the unfavourable trend and progress of the burden of cancer disease in Balkan countries and emphasising the need to increase the national coverage rates of the target population groups in the quality of cancer prevention, screening, and treatment. Secondly, the study extends knowledge in several respects by providing a detailed subgroup analysis according to the type of cancer and per country over the three decades and by 2030. Thirdly, study data have critical implications for the debate about the potential benefits and costs of cancer prevention and control strategies in Balkan countries. Moreover, to address the challenges of the rising burden of cancer at the country level, our study advocates identifying high-risk individuals (given the existence of discrepant screening recommendations (e.g., for colon and rectum cancer screening [46]) for tailored screening uptake and their further integration in population-based health programs, despite it might raise demand for resources.

Several limitations of our study also need to be noted. Firstly, we did not include information on other factors that might influence the disease burden, such as risk factors. The main limitation of our study is that it used temporal data, which can be susceptible to changes in case definitions. Therefore, a careful comparison is necessary since possible differences in the estimated values can be found in other studies due to case definitions and methods used for previous assessments [11, 47].

However, to our knowledge, this is the first study that examined the estimated and forecasted burden of breast, cervical and colon and rectum cancers in Balkan countries for over four decades. However, during the studied period some of the observed countries went through difficult periods, such as wars and migrations, affecting their age and sex population structures and size, which in turn influence the overall cancer burden. We have not analysed the age patterns of the disease burden, and further studies should focus on the in-depth exploration of the factors associated with the burden of these cancers, such as socio-demographic, socio-economic, and lifestyle characteristics. We used the specific rates rather than the standardised rates established in GBD-2019 since the standard world population is younger than the Balkan countries’ population. Nonetheless, in our approach, neither the focus on the progress of cancer burden per country was affected, nor the opportunity to highlight the best and the worst country performers was omitted. Also, our forecasting method only examined time trends of estimates of the YLL, YLD, and DALYs rates without assessing the possible influence of any other factors. More precisely, our forecasts are based on the assumptions that the country-specific policy and practice in health and care, as well as that environmental, behavioural, metabolic and other risk factors such as socioeconomic and demographic structure, will be similar to the situation in the period 1990–2019, i.e., that impact of those factors will not change significantly from 2019 to 2030. In that regard, the effect of any achievement in policy in practice or health promotion in the period from 2019–2030 might reflect in declining the undesirable forecasted trends. On the contrary, the worsening of the country-specific policy and practice regarding these cancers (including health care, environmental risks and population risk behaviour) and the socioeconomic and demographic situation might show the progress of the unwanted forecasted trends. Additionally, we described the burden of breast, cervical, and colorectal cancers in Balkan countries in the period before the COVID-19 pandemic. Assessing the impact of the COVID-19 pandemic on cancer morbidity and mortality will be critical for combating the cancer burden, as the pandemic likely delayed the reduction of health losses from breast, cervical, and colon and rectum cancers in Balkan countries.

## Conclusion

In the Balkan region, the highest incidence and DALYs rates in the study period were for colon and rectum, and breast cancers. Most of the DALYs burden for all three diseases was due to premature mortality. The forecast of YLL rates for colon and rectum cancer does not show a decline by 2030 in the Balkan region. Albania had the lowest incidence and burden of breast, cervical, and colon and rectum cancers among all the Balkan countries in 2019 and is expected to remain the country with the lowest burden of breast and colon and rectum cancers in 2030. The lowest DALY for cervical cancer in 2030 is forecasted for Slovenia, which first introduced obligatory population-based screening. International collaborative action and multistakeholder best practices are needed to remove numerous country-specific barriers to cancer early detection and quality and care continuum.

Future studies should focus on the risk factors for preventable cancers in the countries with the highest burden and examine the possibilities for establishing preventive interventions. The awareness of the importance of screening, access to screening, and coverage must be improved, along with the timely treatment and care continuum, to help countries prevent the enormous losses of healthy lives and premature mortality. This can only be achieved through the broader participation of all stakeholders, including the general public, policymakers, public health professionals and clinicians.

### Supplementary Information

**Additional file 1: Supplemental file 1.** Incidence of breast cancer per 100,000 population in the period 1990-2019, estimates with 95% uncertainty interval per country in the Balkan region, and per cent change 2019 vs 1990. **Supplemental file 2.** Incidence of cervical cancer per 100,000 population in the period 1990-2019, estimates with 95% uncertainty interval per country in the Balkan region, and per cent change 2019 vs 1990. **Supplemental file 3.** Incidence of colon and rectum cancer per 100,000 population in the period 1990-2019, estimates with 95% uncertainty interval per country in the Balkan region, and per cent change 2019 vs 1990. **Supplemental file 4.** Years lived with disability due to breast cancer per 100,000 population in the period 1990-2019, estimates with 95% uncertainty interval per country in the Balkan region, and per cent change 2019 vs 1990. **Supplemental file 5.** Years lived with disability due to cervical cancer per 100,000 population in the period 1990-2019, estimates with 95% uncertainty interval per country in the Balkan region, and per cent change 2019 vs 1990. **Supplemental file 6.** Years lived with disability due to colon and rectum cancer per 100,000 population in the period 1990-2019, estimates with 95% uncertainty interval per country in the Balkan region, and per cent change 2019 vs 1990. **Supplemental file 7.** Years of life lost due to breast cancer per 100,000 population in the period 1990-2019, estimates with 95% uncertainty interval per country in the Balkan region, and per cent change 2019 vs 1990. **Supplemental file 8.** Years of life lost due to cervical cancer per 100,000 population in the period 1990-2019, estimates with 95% uncertainty interval per country in the Balkan region, and per cent change 2019 vs 1990. **Supplemental file 9.** Years of life lost due to colon and rectum cancer per 100,000 population in the period 1990-2019, estimates with 95% uncertainty interval per country in the Balkan region, and per cent change 2019 vs 1990. **Supplemental file 10.** Disability-adjusted life years due to breast cancer per 100,000 population in the period 1990-2019, estimates with 95% uncertainty interval per country in the Balkan region, and per cent change 2019 vs 1990. **Supplemental file 11.** Disability-adjusted life years due to cervical cancer per 100,000 population in the period 1990-2019, estimates with 95% uncertainty interval per country in the Balkan region, and per cent change 2019 vs 1990. **Supplemental file 12.** Disability-adjusted life years due to colon and rectum cancer per 100,000 population in the period 1990-2019, estimates with 95% uncertainty interval per country in the Balkan region, and per cent change 2019 vs 1990.

## Data Availability

This was the secondary analysis of the data from the global burden of Disease study of the Institute of Health metrics and Evaluation.
