# Supplementary material for: The burden of breast, cervical, and colon and rectum cancer in the Balkan countries, 1990–2019 and forecast to 2030
Source: Arch Public Health. 2023 Aug 24;81:156. doi: 10.1186/s13690-023-01137-9 (PMC10464494; doi:10.1186/s13690-023-01137-9)
Supplement: Supplementary file 1 — Additional file 1: Supplemental file 1. Incidence of breast cancer per 100,000 population in the period 1990-2019, estimates with 95% uncertainty interval per country in the Balkan region, and per cent change 2019 vs 1990. Supplemental file 2. Incidence of cervical cancer per 100,000 population in the period 1990-2019, estimates with 95% uncertainty interval per country in the Balkan region, and per cent change 2019 vs 1990. Supplemental file 3. Incidence of colon and rectum cancer per 100,000 population in the period 1990-2019, estimates with 95% uncertainty interval per country in the Balkan region, and per cent change 2019 vs 1990. Supplemental file 4. Years lived with disability due to breast cancer per 100,000 population in the period 1990-2019, estimates with 95% uncertainty interval per country in the Balkan region, and per cent change 2019 vs 1990. Supplemental file 5. Years lived with disability due to cervical cancer per 100,000 population in the period 1990-2019, estimates with 95% uncertainty interval per country in the Balkan region, and per cent change 2019 vs 1990. Supplemental file 6. Years lived with disability due to colon and rectum cancer per 100,000 population in the period 1990-2019, estimates with 95% uncertainty interval per country in the Balkan region, and per cent change 2019 vs 1990. Supplemental file 7. Years of life lost due to breast cancer per 100,000 population in the period 1990-2019, estimates with 95% uncertainty interval per country in the Balkan region, and per cent change 2019 vs 1990. Supplemental file 8. Years of life lost due to cervical cancer per 100,000 population in the period 1990-2019, estimates with 95% uncertainty interval per country in the Balkan region, and per cent change 2019 vs 1990. Supplemental file 9. Years of life lost due to colon and rectum cancer per 100,000 population in the period 1990-2019, estimates with 95% uncertainty interval per country in the Balkan region, and per cent change 2019 vs 1990. Supp [file 13690_2023_1137_MOESM1_ESM.docx]

Supplemental file 1. Incidence of breast cancer per 100,000 population in the period 1990-2019, estimates with 95% uncertainty interval per country in the Balkan region, and per cent change 2019 vs 1990

| Country | 1990 | 1995 | 2000 | 2005 | 2010 | 2015 | 2019 | 2019 vs 1990 (%) |
| --- | --- | --- | --- | --- | --- | --- | --- | --- |
| Albania | 6.83 (6.23-7.48) | 8.64 (7.89-9.45) | 12.53 (11.44-13.61) | 18.31 (16.80-19.88) | 22.48 (18.66-26.78) | 25.70 (18.69-33.38) | 28.10 (20.41-37.90) | 311.42 |
| Bosnia and Herzegovina | 14.25 (13.03-15.68) | 18.47 (14.42-23.74) | 22.94 (19.02-26.82) | 32.53 (28.29-37.29) | 39.77 (35.36-44.39) | 46.34 (41.90-50.60) | 49.54 (37.97-63.78) | 247.65 |
| Bulgaria | 38.96 (35.83-42.06) | 33.39 (30.90-35.83) | 37.15 (34.35-39.92) | 52.64 (48.69-56.54) | 64.61 (59.20-69.99) | 73.53 (66.98-80.13) | 73.86 (57.73-93.47) | 89.58 |
| Croatia | 45.59 (41.49-49.76) | 48.86 (44.95-53.03) | 49.21 (45.44-53.32) | 55.79 (52.40-58.97) | 65.65 (61.08-70.43) | 74.88 (68.53-81.03) | 67.44 (52.12-85.83) | 47.93 |
| Greece | 50.95 (47.54-54.51) | 57.89 (53.95-62.18) | 64.08 (59.43-68.62) | 72.92 (67.40-78.12) | 77.16 (71.34-83.38) | 77.85 (71.79-84.46) | 85.13 (65.97-108.93) | 67.09 |
| Montenegro | 33.66 (27.70-42.23) | 38.05 (32.20-45.13) | 45.54 (40.76-51.51) | 52.62 (46.97-58.72) | 60.52 (53.05-68.39) | 65.12 (55.66-76.28) | 66.58 (53.05-81.96) | 97.80 |
| North Macedonia | 24.69 (21.93-27.69) | 30.44 (27.72-33.24) | 37.94 (34.50-41.53) | 41.95 (38.27-46.14) | 49.28 (44.64-54.51) | 52.08 (45.79-59.30) | 54.24 (40.96-70.27) | 119.68 |
| Republic of Moldova | 24.15 (22.64-25.77) | 26.22 (24.08-28.28) | 24.54 (22.92-26.21) | 29.14 (27.29-31.18) | 32.86 (30.65-35.28) | 36.56 (33.88-39.13) | 33.74 (28.52-39.82) | 39.71 |
| Romania | 21.41 (20.32-22.65) | 25.81 (24.44-27-22) | 29.04 (27.40-30.70) | 33.77 (31.76-35.83) | 37.64 (35.42-39.80) | 42.73 (40.15-45.36) | 45.49 (36.85-55.28) | 112.47 |
| Serbia | 34.65 (30.50-39.59) | 42.49 (38.75-46.49) | 48.89 (46.01-52.21) | 56.55 (53.15-60.07) | 64.39 (60.18-68.55) | 69.71 (64.65-75.35) | 71.49 (40.96-70.27) | 106.32 |
| Slovenia | 44.13 (32.68-57.28) | 47.89 (38.61-58.95) | 55.63 (51.60-59.94) | 59.57 (54.79-64.37) | 60.13 (54.97-65.66) | 59.61 (53.90-66.03) | 59.43 (45.64-78.99) | 34.67 |

Supplemental file 2. Incidence of cervical cancer per 100,000 population in the period 1990-2019, estimates with 95% uncertainty interval per country in the Balkan region, and per cent change 2019 vs 1990

| Country | 1990 | 1995 | 2000 | 2005 | 2010 | 2015 | 2019 | 2019 vs 1990 (%) |
| --- | --- | --- | --- | --- | --- | --- | --- | --- |
| Albania | 3.12 (4.21-2.72) | 3.16 (2.76-4.32) | 3.77 (3.34-4.74) | 4.62 (3.86-5.31) | 4.67 (3.68-5.74) | 5.01 (3.48-6.72) | 5.16 (3.57-7.14) | 65.38 |
| Bosnia and Herzegovina | 6.57 (5.76-7.88) | 8.30 (6.29-11.16) | 7.70 (6.36-9.43) | 8.89 (6.86-10.22) | 9.50 (7.12-10.79) | 10.36 (7.27-11.71) | 10.19 (6.84-13.31) | 55.10 |
| Bulgaria | 11.80 (10.44-13.31) | 10.27 (9.31-12.84) | 11.62 (10.51-13.37) | 14.17 (11.93-15.43) | 16.11 (11.11-17.82) | 16.78 (11.20-21.99) | 16.78 (11.20-21.99) | 42.20 |
| Croatia | 13.51 (11.15-15.21) | 12.94 (11.36-14.34) | 10.42 (9.40-12.26) | 10.05 (9.19-11.62) | 10.88 (9.75-11.90) | 11.30 (9.85-12.49) | 10.01 (7.47-13.13) | -25.91 |
| Greece | 7.25 (6.52-7.90) | 6.67 (6.05-7.26) | 6.28 (5.74-6.90) | 5.99 (5.49-6.78) | 6.31 (5.75-6.99) | 6.29 (5.70-7.19) | 6.67 (5.11-8.72) | -8.00 |
| Montenegro | 7.22 (5.86-9.29) | 8.09 (6.76-10.14) | 9.73 (8.14-11.40) | 10.07 (8.43-11.75) | 9.45 (8.02-11.24) | 9.41 (8.01-11.33) | 9.13 (7.25-11.62) | 26.45 |
| North Macedonia | 7.52 (6.54-9.85) | 9.71 (8.07-11.15) | 12.02 (8.76-13.59) | 10.52 (8.38-11.99) | 9.94 (8.44-11.64) | 9.94 (8.31-11.76) | 9.90 (7.08-13.30) | 31.65 |
| Republic of Moldova | 11.87 (9.99-12.93) | 11.53 (10.02-13.66) | 10.39 (9.59-12.25) | 12.14 (11.07-13.36) | 12.69 (11.33-13.81) | 12.57 (10.50-13.65) | 10.81 (8.55-13.36) | -8.93 |
| Romania | 16.95 (15.70-18.13) | 19.25 (16.99-20.77) | 21.02 (16.46-22.72) | 22.17 (16.14-23.87) | 21.44 (15.35-23.15) | 20.75 (14.62-22.48) | 20.44 (14.22-25.81) | 20.59 |
| Serbia | 15.39 (12.35-18.04) | 17.74 (13.95-20.46) | 18.06 (14.02-19.83) | 18.05 (13.87-19.87) | 16.73 (13.01-18.51) | 16.20 (12.42-18.00) | 15.67 (11.41-20.55) | 1.82 |
| Slovenia | 10.69 (7.81-14.35) | 10.49 (8.16-13.33) | 10.22 (9.06-11.49) | 9.03 (8.06-10.08) | 8.20 (7.26-9.29) | 7.91 (6.78-9.18) | 7.63 (5.52-10.63) | -28.62 |

Supplemental file 3. Incidence of colon and rectum cancer per 100,000 population in the period 1990-2019, estimates with 95% uncertainty interval per country in the Balkan region, and per cent change 2019 vs 1990

| Country | 1990 | 1995 | 2000 | 2005 | 2010 | 2015 | 2019 | 2019 vs 1990 (%) |
| --- | --- | --- | --- | --- | --- | --- | --- | --- |
| Albania | 6.63 (6.17-7.10) | 6.63 (6.16-7.13) | 8.18 (7.65-8.68) | 11.57 (10.82-12.33) | 14.57 (12.48-16.99) | 19.62 (14.68-24.85) | 23.15 (17.29-30.43) | 249.17 |
| Bosnia and Herzegovina | 15.92 (15.06-16.87) | 20.20 (16.22-25.14) | 26.73 (23.54-29.66) | 39.20 (35.79-42.90) | 47.54 (44.09-51.31) | 56.10 (52.00-59.75) | 62.35 (49.06-77.90) | 291.65 |
| Bulgaria | 40.53 (38.35-42.66) | 33.37 (31.71-35.02) | 39.42 (37.36-41.53) | 60.51 (57.38-63.49) | 75.62 (71.63-79.72) | 84.16 (78.84-89.32) | 89.39 (71.75-110.18) | 120.55 |
| Croatia | 44.98 (42.27-47.88) | 50.72 (47.77-53.41) | 58.29 (54.93-61.72) | 74.23 (71.10-77.31) | 89.84 (85.57-93.88) | 105.26 (98.88-110.65) | 103.79 (82.47-128.83) | 130.75 |
| Greece | 36.99 (35.03-38.83) | 45.82 (43.40-48.08) | 53.77 (50.58-56.55) | 58.89 (55.47-62.13) | 61.62 (57.61-65.19) | 68.99 (64.00-73.00) | 76.45 (60.72-96.16) | 106.68 |
| Montenegro | 21.59 (18.14-24.71) | 22.81(20.54-25.04) | 25.98 (23.95-28.21) | 32.69 (30.55-35.27) | 38.61 (35.26-42.01) | 44.62 (39.26-49.98) | 48.27 (39.50-58.35) | 123.58 |
| North Macedonia | 17.86 (16.31-19.23) | 22.51 (21.27-23.83) | 30.60 (28.92-32.29) | 36.82 (34.82-38.84) | 44.33 (41.72-46.93) | 48.85 (45.29-52.47) | 52.71 (41.73-65.78) | 195.13 |
| Republic of Moldova | 25.03 (24.01-26.22) | 26.69 (25.62-28.03) | 22.25 (21.32-23.10) | 30.97 (29.51-32.44) | 41.34 (39.49-43.25) | 48.08 (45.76-50.38) | 45.85 (39.91-52.25) | 183.18 |
| Romania | 20.78 (19.99-21.55) | 28.98 (27.82-30.15) | 30.72 (29.47-31.99) | 39.72 (38.17-41.30) | 51.25 (49.06-53.39) | 60.48 (57.60-63.23) | 67.78 (55.86-80.79) | 226.18 |
| Serbia | 32.73 (26.86-36.64) | 37.79 (33.56-40.92) | 44.74 (42.90-46.73) | 56.97 (54.70-59.52) | 67.91 (64.64-71.16) | 75.13 (71.06-79.28) | 77.62 (62.33-96.56) | 137.15 |
| Slovenia | 40.76 (31.48-51.95) | 48.50 (39.73-58.10) | 58.55 (55.43-61.67) | 68.26 (64.21-71.77) | 78.80 (73.69-83.57) | 78.12 (71.86-84.18) | 83.22 (64.91-107.10) | 104.17 |

Supplemental file 4. Years lived with disability due to breast cancer per 100,000 population in the period 1990-2019, estimates with 95% uncertainty interval per country in the Balkan region, and per cent change 2019 vs 1990

| Country | 1990 | 1995 | 2000 | 2005 | 2010 | 2015 | 2019 | 2019 vs 1990 (%) |
| --- | --- | --- | --- | --- | --- | --- | --- | --- |
| Albania | 5.00  (3.41-6.94) | 6.01  (4.14-8.57) | 8.5  (5.88-11.6) | 12.40  (8.54-17.10) | 15.52  (10.42-21.91) | 17.97  (11.57-26.54) | 19.86 (12.28-29.88) | 297.20 |
| Bosnia and Herzegovina | 14.64  (10.11-20.12) | 12.32  (8.04-17.59) | 15.26  (10.45-21.37) | 21.22  (14.75-29.12) | 26.25  (18.35-35.99) | 30.58  (21.20-41.56) | 32.93 (21.92-46.96) | 230.62 |
| Bulgaria | 16.12  (11.30-21.74) | 24.11  (7.08-33.58) | 26.39  (18.27-36.07) | 36.05  (25.17-48.21) | 43.59  (30.39-59.36) | 49.76  (34.37-66.70) | 50.80 (33.31-72.54) | 86.01 |
| Croatia | 27.31  (19.05-37.4) | 33.62  (23.57-45.97) | 34.21  (24.13-46.74) | 38.43  (27.25-52.1) | 44.61  (31.78-60.17) | 50.37  (35.70-67.80) | 45.98 (30.35-64.60) | 48.23 |
| Greece | 31.02  (21.79-41.53) | 43.32  (30.32-59.08) | 47.64  (33.41-65.42) | 54.00  (37.69-74.07) | 57.95  (40.03-78.70) | 59.19  (41.15-81.63) | 64.38 (43.24-92.61) | 61.80 |
| Montenegro | 29.58  (19.06-42.92) | 25.90  (17.58-36.15) | 30.82  (21.51-42.33) | 35.76  (24.71-48.98) | 41.25  (28.80-56.79) | 44.53  (31.04-61.99) | 45.85 (30.63-64.51) | 97.63 |
| North Macedonia | 23.21  (5.43-33.77) | 20.29  (14.14-27.65) | 25.22  (17.74-34.55) | 27.93  (19.53-38.28) | 32.95  (22.81-44.59) | 34.92  (23.98-48.73) | 36.54 (23.36-53.99) | 118.02 |
| Republic of Moldova | 9.96  (6.94-13.71) | 17.42  (12.28-23.28) | 16.77  (11.68-22.98) | 19.64  (13.65-27.03) | 21.97  (15.31-30.06) | 24.57  (17.46-33.31) | 23.16 (15.59-32.34) | 43.67 |
| Romania | 39.79  (27.64-54.09) | 17.02  (12.05-22.91) | 19.30  (13.54-25.83) | 22.45  (15.73-30.54) | 25.28  (17.72-34.54) | 28.71  (20.18-39.07) | 30.76 (20.87-42.97) | 110.11 |
| Serbia | 16.76  (11.29-23.08) | 27.88  (19.29-38.46) | 32.43  (22.66-44.18) | 37.50  (26.29-49.98) | 43.01  (30.51-58.23) | 46.84  (33.15-63.07) | 48.14 (31.82-69.39) | 106.26 |
| Slovenia | 23.34  (15.74-32.12) | 32.33  (21.60-45.87) | 37.44  (26.31-51.40) | 40.29  (28.29-54.88) | 41.08  (28.47-55.86) | 41.19  (28.16-56.26) | 41.30 (26.52-59.76) | 39.62 |

Supplemental file 5. Years lived with disability due to cervical cancer per 100,000 population in the period 1990-2019, estimates with 95% uncertainty interval per country in the Balkan region, and per cent change 2019 vs 1990

| Country | 1990 | 1995 | 2000 | 2005 | 2010 | 2015 | 2019 | 2019 vs 1990 (%) |
| --- | --- | --- | --- | --- | --- | --- | --- | --- |
| Albania | 1.42  (0.95-2.04) | 1.46  (0.99-2.13) | 1.75  (1.21-2.47) | 2.14  (1.43-2.97) | 2.16  (1.44-3.09) | 2.31  (1.39-3.53) | 2.37 (1.43-3.62) | 66.90 |
| Bosnia and Herzegovina | 2.8  (1.86-3.91) | 3.46  (2.15-5.27) | 3.32  (2.2-4.72) | 3.85  (2.52-5.33) | 4.17  (2.71-5.7) | 4.5  (2.82-6.10) | 4.45 (2.71-6.69) | 58.93 |
| Bulgaria | 5.24  (3.58-7.03) | 4.56  (3.05-6.34) | 5.15  (3.52-7.07) | 6.24  (4.11-8.53) | 7.13  (4.33-9.83) | 7.87  (4.64-10.66) | 7.51 (4.17-11.26) | 43.32 |
| Croatia | 6.32  (4.29-8.59) | 6.08  (4.15-8.25) | 4.99  (3.44-6.86) | 4.84  (3.34-6.69) | 5.16  (3.52-7.00) | 5.38  (3.71-7.25) | 4.81 (3.03-7.26) | -23.89 |
| Greece | 3.23  (2.24-4.4) | 2.99  (2.07-4.09) | 2.81  (1.97-3.81) | 2.72  (1.91-3.65) | 2.89  (1.99-3.92) | 2.87  (2.00-3.91) | 2.99 (1.90-4.43) | -7.43 |
| Montenegro | 3.43  (2.17-5.1) | 3.75  (2.43-5.43) | 4.46  (2.9-6.21) | 4.58  (3.00-6.36) | 4.29  (2.86-6.05) | 4.33  (2.93-6.07) | 4.28 (2.84-6.26) | 24.78 |
| North Macedonia | 3.32  (2.15-4.72) | 4.26  (2.83-5.91) | 5.23  (3.23-7.23) | 4.56  (3.00-6.3) | 4.40  (2.89-6.24) | 4.39  (2.98-6.13) | 4.36 (2.69-6.53) | 31.33 |
| Republic of Moldova | 4.75  (3.22-6.42) | 4.67  (3.19-6.37) | 4.37  (2.96-6.08) | 5.12  (3.55-6.93) | 5.38  (3.61-7.32) | 5.39  (3.63-7.39) | 4.67 (2.93-6.77) | -1.68 |
| Romania | 7.14  (4.9-9.55) | 8.21  (5.55-10.98) | 9.08  (5.9-12.51) | 9.58  (6.20-12.87) | 9.25  (6.02-12.73) | 8.89  (5.64-12.09) | 8.81 (5.37-12.85) | 23.39 |
| Serbia | 6.59  (4.29-9.4) | 7.64  (5.07-10.49) | 7.85  (5.21-10.85) | 7.78  (5.28-10.7) | 7.28  (4.82-10.04) | 7.1  (4.66-9.57) | 6.87 (4.24-10.20) | 4.25 |
| Slovenia | 4.93  (3.05-7.51) | 4.9  (3.18-7.16) | 4.86  (3.27-6.65) | 4.33  (2.96-6.01) | 3.98  (2.72-5.42) | 3.88  (2.61-5.36)_ | 3.74 (2.31-5.73) | -24.24 |

Supplemental file 6. Years lived with disability due to colon and rectum cancer per 100,000 population in the period 1990-2019, estimates with 95% uncertainty interval per country in the Balkan region, and per cent change 2019 vs 1990

| Country | 1990 | 1995 | 2000 | 2005 | 2010 | 2015 | 2019 | 2019 vs 1990 (%) |
| --- | --- | --- | --- | --- | --- | --- | --- | --- |
| Albania | 2.91  (2.12-3.78) | 3  (2.17-3.99) | 3.69  (2.66-4.87) | 5.22  (3.77-6.91) | 6.71  (4.74-9.02) | 9.04  (6.03-13.01) | 10.67 (6.86-15.53) | 266.67 |
| Bosnia and Herzegovina | 6.77  (4.9-9.15) | 8.37  (5.65-11.82) | 11.38  (8.16-15.15) | 16.59  (11.95-21.79) | 20.47  (14.99-26.47) | 24.06  (17.62-31.01) | 26.93 (18.49-37.77) | 297.78 |
| Bulgaria | 17.64  (13.04-22.78) | 14.71  (10.76-18.92) | 17.39  (12.88-22.61) | 26.15  (19.25-33.77) | 32.68  (24.16-42.12) | 37.05  (27.24-47.99) | 39.93 (27.35-56.18) | 126.36 |
| Croatia | 19.83  (14.37-25.5) | 22.58  (16.74-29.34) | 26.37  (19.15-33.91) | 33.46  (24.39-42.86) | 40.8  (30.12-52.44) | 48.13  (35.46-62.4) | 47.72 (32.97-47.72) | 140.65 |
| Greece | 18.66  (13.66-24.23) | 22.58  (16.44-29.27) | 26.59  (19.49-34.63) | 29.52  (1.57-38.71) | 31.41  (22.94-41.22) | 34.78  (25.55-44.86) | 38.10 (26.34-52.79) | 104.18 |
| Montenegro | 9.81  (6.78-13.03) | 10.25  (7.35-13.42) | 11.62  (8.39-15.21) | 14.6  (10.57-19.31) | 17.49  (12.51-22.95) | 20.32  (14.57-26.32) | 22.22 (15.27-29.98) | 126.50 |
| North Macedonia | 7.7  (5.41-10.15) | 9.57  (6.94-12.52) | 13.04  (9.57-16.92) | 15.83  (11.41-20.45) | 19.48  (14.11-25.45) | 21.64  (15.89-27.63) | 23.57 (16.02-33.11) | 206.10 |
| Republic of Moldova | 10.57  (7.73-13.92) | 11.11  (8.05-14.25) | 9.71  (7.031-2.76) | 13.37  (9.62-17.27) | 17.76  (12.85-22.93) | 21.18  (15.5-27.65) | 20.65 (14.85-27.38) | 95.36 |
| Romania | 8.94  (6.42-11.64) | 12.24  (9.02-15.91) | 13.33  (9.75-17.26) | 17.33  (12.77-22.28) | 22.7  (16.78-29.00) | 26.99  (19.87-34.9) | 30.65 (21.78-41.79) | 242.84 |
| Serbia | 13.72  (9.49-18.16) | 15.99  (11.22-20.73) | 19.13  (13.98-24.48) | 24.33  (17.67-31.4) | 29.88  (21.82-39.22) | 33.62  (24.64-43.69) | 34.70 (23.56-48.14) | 152.92 |
| Slovenia | 17.71  (11.89-25.18) | 21.31  (5.04-28.24) | 26.02  (19.17-33.72) | 30.86  (22.62-39.74) | 36.46  (26.42-46.83) | 36.71  (26.29-48.18) | 39.16 (25.94-55.77) | 121.12 |

Supplemental file 7. Years of life lost due to breast cancer per 100,000 population in the period 1990-2019, estimates with 95% uncertainty interval per country in the Balkan region, and per cent change 2019 vs 1990

| Country | 1990 | 1995 | 2000 | 2005 | 2010 | 2015 | 2019 | 2019 vs 1990 (%) |
| --- | --- | --- | --- | --- | --- | --- | --- | --- |
| Albania | 109.94 (101.24-119.82) | 119.22 (109.59-129.58) | 160.68 (148.67-172.07) | 210.17 (195.25-225.07) | 226.14  (189.22-269.29) | 241.09  (176.72-313.12) | 251.38 (185.33-340.23) | 128.65 |
| Bosnia and Herzegovina | 223.13 (205.00-243.42) | 290.51 (227.42-372.98) | 292.79 (245.08-340.62) | 367.92 (324.08-418.83) | 415.84  (374.56-461.30) | 466.67  (424.96-505.40) | 480.74 (372.77-609.93) | 115.45 |
| Bulgaria | 427.86 (397.86-458.49) | 359.89 (336.76-384.54) | 374.84 (354.74-400.57) | 499.35  (468.22-531.25) | 581.18  (540.31-623.77) | 611.66  (565.54-659.09) | 586.06 (459.01-739.73) | 36.97 |
| Croatia | 468.74 (432.13-508.17) | 486.75 (453.43-523.84) | 448.13 (418.81-479.88) | 466.38  (442.26-488.25) | 507.25  (479.93-534.04) | 535.32  (499.58-569.71) | 467.31 (358.65-589.86) | -0.31 |
| Greece | 440.10 (421.86-458.27) | 447.22 (427.86-465.32) | 447.95 (426.82-466.48) | 533.56  (480.16-592.18) | 462.27  (437.22-484.55) | 472.22  (440.78-498.30) | 510.13 (468.72-548.63) | 15.91 |
| Montenegro | 369.04 (302.00-457.44) | 414.99 (352.47-489.61) | 493.61 (442.52-548.92) | 533.56  (480.16-592.18) | 556.20  (495.36-627.75) | 560.00  (483.14-649.41) | 552.44 (440.74-679.92) | 49.70 |
| North Macedonia | 378.28 (337.95-421.76) | 422.88 (389.53-459.64) | 473.53 (438.17-514.12) | 479.97  (442.08-519.05) | 507.63  (463.78-554.65) | 513.42  (453.20-580.86) | 517.42 (393.05-661.17) | 36.78 |
| Republic of Moldova | 377.06 (357.75-397.41) | 418.00 (387.28-443.29) | 353.62 (335.89-372.38) | 397.03  (376.20-418.85) | 426.33  (404.66-449.31) | 426.90  (403.25-452.47) | 367.49 (312.86-434.87) | -2.54 |
| Romania | 331.66 (318.03-347.35) | 371.94 (357.38-386.54) | 372.15 (357.65-386.25) | 403.85  (387.93-421.03) | 417.80  (400.54-434.25) | 441.70  (422.42-461.70) | 448.13 (363.03-545.98) | 35.12 |
| Serbia | 486.56 (430.92-549.41) | 556.68 (512.99-606.08) | 587.27 (558.46-619.19) | 628.55  (596.73-661.34) | 626.18  (594.90-655.75) | 624.12  (588.10-662.92) | 622.70 (492.48-788.14) | 27.98 |
| Slovenia | 466.96 (350.26-613.95) | 479.93 (388.16-588.01) | 518.90 (488.28-549.88) | 503.10  (469.64-536.35) | 456.31  (421.30-490.87) | 417.64  (377.99-457.45) | 408.89 (314.89-541.23) | -12.44 |

Supplemental file 8. Years of life lost due to cervical cancer per 100,000 population in the period 1990-2019, estimates with 95% uncertainty interval per country in the Balkan region, and per cent change 2019 vs 1990

| Country | 1990 | 1995 | 2000 | 2005 | 2010 | 2015 | 2019 | 2019 vs 1990 (%) |
| --- | --- | --- | --- | --- | --- | --- | --- | --- |
| Albania | 46.96  (41.31-64.62) | 44.40  (38.92-61.02) | 52.55  (46.70-65.94) | 62.65  (52.00-71.48) | 60.72  (47.37-74.40) | 63.52  (44.56-85.49) | 64.33 (44.18-89.48) | 36.99 |
| Bosnia and Herzegovina | 104.46  (91.68-125.23) | 135.81  (103.18-183.04) | 115.09  (94.74-141.23) | 126.86  (97.98-145.31) | 131.83  (97.44-148.84) | 140.75  (98.71-158.75) | 136.95 (93.28-178.98) | 31.10 |
| Bulgaria | 166.57  (149.63-186.26) | 144.51  (132.14-184.24) | 162.01  (147.36-189.37) | 196.57  (169.09-212.97) | 218.38  (152.70-240.04) | 231.39  (144.84-258.48) | 215.06 (147.33-282.68) | 29.11 |
| Croatia | 140.79  (117.40-156.44) | 131.46  (117.60-144.47) | 101.24  (91.92-119.11) | 96.15  (88.68-109.39) | 101.09  (91.87-109.35) | 101.47  (88.71-111.91) | 88.72 (66.51-114.79) | -36.98 |
| Greece | 88.07  (79.35-94.37) | 76.37  (70.43-81.60) | 69.89  (64.42-75.25) | 63.92  (59.33-72.42) | 66.82  (61.55-73.31) | 68.32  (62.43-76.72) | 71.73 (64.79-80.85) | -18.55 |
| Montenegro | 90.61  (74.28-117.35) | 104.76  (88.46-130.97) | 127.13  (109.12-150.07) | 130.29  (111.56-151.73) | 118.90  (102.22-140.98) | 114.10  (98.16-136.97) | 109.04 (86.53-136.43) | 20.34 |
| North Macedonia | 114.46  (99.68-153.02) | 143.48  (121.70-163.94) | 173.85  (126.26-194.72) | 149.62  (118.63-169.91) | 134.74  (113.92-157.25) | 132.23  (110.97-155.55) | 129.70 (93.14-173.08) | 13.31 |
| Republic of Moldova | 204.94  (174.34-221.29) | 200.49  (176.07-238.61) | 171.28  (159.45-205.34) | 196.03  (180.81-213.95) | 201.93  (182.04-217.90) | 190.43  (163.30-205.80) | 159.35 (128.88-195.62) | -22.25 |
| Romania | 265.45  (249.45-283.46) | 294.05  (262.77-311.60) | 307.28  (245.16-327.41) | 318.86  (236.06-338.20) | 301.06  (211.98-320.39) | 284.97  (195.61-305.14) | 274.66 (189.36-347.23) | 3.47 |
| Serbia | 229.15  (185.51-265.11) | 256.69  (207.41-291.48) | 252.30  (197.86-276.11) | 249.07  (189.20-270.49) | 219.60  (170.81-240.29) | 203.95  (156.80-225.02) | 194.99 (142.19-254.76) | -14.91 |
| Slovenia | 117.47  (85.90-158.00) | 111.93  (87.22-140.22) | 105.30  (93.66-115.96) | 88.58  (80.16-97.41) | 77.05  (69.00-86.71) | 71.79  (62.12-82.04) | 68.56 (50.65-94.11) | -41.46 |

Supplemental file 9. Years of life lost due to colon and rectum cancer per 100,000 population in the period 1990-2019, estimates with 95% uncertainty interval per country in the Balkan region, and per cent change 2019 vs 1990

| Country | 1990 | 1995 | 2000 | 2005 | 2010 | 2015 | 2019 | 2019 vs 1990 (%) |
| --- | --- | --- | --- | --- | --- | --- | --- | --- |
| Albania | 132.34  (123.78-141.41) | 120.69  (112.76-129.18) | 141.13  (133.23-149.39) | 182.85  (172.61-193.24) | 206.45  (175.44-241.59) | 259.12  (192.84-328.42) | 291.68 (217.41-383.50) | 120.40 |
| Bosnia and Herzegovina | 308.57  (292.18-326.22) | 392.16  (312.65-490.33) | 450.61  (396.85-499.97) | 600.46  (551.28-651.85) | 679.82  (631.96-730.58) | 771.00  (720.02-816.57) | 829.10 (653.26-1040.25) | 168.69 |
| Bulgaria | 662.43  (628.02-695.59) | 533.33  (508.61-558.94) | 583.79  (552.51-612.61) | 841.50  (800.85-880.21) | 1,003.41  (954.42-1,053.51) | 1,058.45  (997.61-1,117.35) | 1085.49 (866.39-1341.75) | 63.86 |
| Croatia | 619.74  (584.09-657.39) | 684.47  (645.49-719.77) | 726.40  (687.81-766.38) | 858.44  (824.21-888.89) | 960.85  (920.41-995.46) | 1,043.65  (989.74-1,088.93) | 996.60 (789.01-1244.96) | 60.81 |
| Greece | 389.31  (372.04-404.30) | 442.42  (424.41-458.44) | 479.39  (457.10-497.45) | 487.39  (465.79-506.60) | 492.54  (466.58-513.73) | 550.43  (517.92-575.95) | 604.21 (559.92-643.23) | 55.20 |
| Montenegro | 317.36  (267.51-362.51) | 337.43  (303.37-370.11) | 385.28  (356.30-415.38) | 457.75  (428.68-490.42) | 496.25  (456.66-539.05) | 540.04  (480.11-600.00) | 568.33 (467.44-643.23) | 157.61 |
| North Macedonia | 333.30  (304.95-357.87) | 398.37  (376.88-421.92) | 503.33  (475.88-530.70) | 568.37  (538.60-599.34) | 632.63  (598.48-667.39) | 671.98  (625.59-718.13) | 705.53 (555.57-886.31) | 111.68 |
| Republic of Moldova | 475.94  (457.58-497.00) | 513.04  (492.05-540.51) | 398.21  (383.26-412.99) | 531.47  (510.38-555.92) | 682.76  (654.68-712.22) | 730.40  (700.70-761.80) | 656.02 (570.78-746.72) | 19.41 |
| Romania | 381.89  (368.43-395.17) | 504.26  (486.23-521.50) | 480.47  (460.09-497.71) | 573.59  (554.82-593.06) | 686.84  (662.28-711.89) | 759.22  (727.48-786.52) | 817.55 (669.65-987.32) | 144.17 |
| Serbia | 582.53  (478.52-652.69) | 643.93  (577.85-696.47) | 708.19  (681.84-737.07) | 838.10  (808.34-869.57) | 891.67  (855.94-929.62) | 922.22  (879.59-966.03) | 932.46 (742.73-1160.30) | 12.62 |
| Slovenia | 564.18  (434.14-729.93) | 644.17  (527.54-777.73) | 726.79  (692.85-760.21) | 765.41  (725.39-801.04) | 797.32  (747.59-840.37) | 741.80  (685.37-792.86) | 771.21 (598.65-994.53) | 36.70 |

Supplemental file 10. Disability-adjusted life years due to breast cancer per 100,000 population in the period 1990-2019, estimates with 95% uncertainty interval per country in the Balkan region, and per cent change 2019 vs 1990

| Country | 1990 | 1995 | 2000 | 2005 | 2010 | 2015 | 2019 | 2019 vs 1990 (%) |
| --- | --- | --- | --- | --- | --- | --- | --- | --- |
| Albania | 114.94 (105.72-125.31) | 125.22 (115.41-136.41) | 169.18 (156.67-182.12) | 222.57 (206.53-239.34) | 241.66 (202.14-287.86) | 259.06 (191.60-334.49) | 271.24 (199.18-366.32) | 135.98 |
| Bosnia and Herzegovina | 233.08 (214.56-253.81) | 302.83 (236.85-389.58) | 308.05 (258.15-359.24) | 389.14 (343.33-443.79) | 442.08 (398.39-491.61) | 497.26 (451.30-537.55) | 513.67 (396.77-659.09) | 120.38 |
| Bulgaria | 455.17 (423.69-488.53) | 383.98 (358.56-410.68) | 401.23 (374.23-430.27) | 535.40 (499.64-571.72) | 624.77 (578.95-670.49) | 661.42 (612.48-713.00) | 636.85 (499.68-806.77) | 39.91 |
| Croatia | 499.76 (462.52-541.04) | 520.37 (484.26-560.73) | 482.35 (448.07-521.28) | 504.81 (476.52-532.44) | 551.87 (521.50-583.53) | 585.69 (544.92-627.92) | 513.29 (398.19-648.19) | 2.71 |
| Greece | 479.89 (456.66-504.76) | 490.54 (465.92-515.10) | 495.59 (469.61-522.31) | 515.54 (485.70-545.74) | 520.22 (486.11-552.31) | 531.40 (492.35-568.13) | 574.51 (525.37-631.90) | 19.72 |
| Montenegro | 392.24 (321.55-485.58) | 440.89 (374.18-520.38) | 524.43 (472.07-585.18) | 569.32 (510.84-632.51) | 597.45 (528.80-677.37) | 604.53 (519.86-700.89) | 598.28 (477.93-734.52) | 52.53 |
| North Macedonia | 395.05 (353.65-439.72) | 443.18 (407.38-480.97) | 498.75 (460.93-543.81) | 507.90 (468.86-550.62) | 540.58 (492.70-590.91) | 548.33 (484.17-620.93) | 553.96 (419.65-710.83) | 40.23 |
| Republic of Moldova | 393.18 (372.52-415.52) | 435.42 (402.43-461.62) | 370.39 (351.04-390.97) | 416.67 (394.86-440.69) | 448.30 (423.67-474.57) | 451.47 (424.85-479.40) | 390.65 (333.50-462.97) | -0.64 |
| Romania | 346.30 (331.41-363.65) | 388.96 (372.09-405.67) | 391.45 (375.51-408.40) | 426.30 (407.90-445.54) | 443.08 (423.25-463.00) | 470.41 (448.59-493.25) | 478.90 (387.45-585.89) | 38.29 |
| Serbia | 509.89 (450.89-577.34) | 584.56 (538.66-636.04) | 619.70 (588.02-653.22) | 666.05 (630.22-703.86) | 669.19 (633.49-703.78) | 670.96 (629.78-716.57) | 670.84 (530.31-847.04) | 31.57 |
| Slovenia | 496.54 (374.16-650.94) | 512.27 (414.67-626.27) | 556.34 (521.00-593.15) | 543.38 (504.59-581.25) | 497.40 (457.94-535.82) | 458.83 (413.56-503.42) | 450.19 (348.30-589.97) | -9.33 |

Supplemental file 11. Disability-adjusted life years due to cervical cancer per 100,000 population in the period 1990-2019, estimates with 95% uncertainty interval per country in the Balkan region, and per cent change 2019 vs 1990

| Country | 1990 | 1995 | 2000 | 2005 | 2010 | 2015 | 2019 | 2019 vs 1990 (%) |
| --- | --- | --- | --- | --- | --- | --- | --- | --- |
| Albania | 48.38 (42.40-66.57) | 45.86 (40.20-62.98) | 54.30 (48.27-68.56) | 64.79 (53.82-74.07) | 62.88 (49.23-77.01) | 65.83 (46.03-88.84) | 66.69 (45.75-92.41) | 37.85 |
| Bosnia and Herzegovina | 107.26 (94.18-128.09) | 139.27 (105.71-187.93) | 118.41 (97.71-145.09) | 130.71 (101.18-149.60) | 135.99 (101.16-153.62) | 145.25 (102.15-163.61) | 141.40 (96.52-185.10) | 31.83 |
| Bulgaria | 171.81 (154.39-192.10) | 149.08 (136.06-189.93) | 167.16 (152.00-195.26) | 202.82 (173.54-219.35) | 225.51 (158.39-248.38) | 239.25 (148.87-266.77) | 222.57 (152.24-291.16) | 29.54 |
| Croatia | 147.11 (123.92-163.55) | 137.55 (122.92-151.62) | 106.23 (96.42-125.07) | 100.99 (93.19-114.29) | 106.25 (96.20-115.27) | 106.85 (93.09-117.82) | 93.53 (70.52-121.44) | -36.42 |
| Greece | 91.29 (82.49-98.22) | 79.36 (73.37-85.01) | 72.70 (67.17-78.29) | 66.63 (61.58-75.39) | 69.71 (64.08-76.23) | 71.19 (64.94-79.75) | 74.72 (67.39-84.12) | -18.15 |
| Montenegro | 94.03 (77.34-121.81) | 108.52 (91.62-136.01) | 131.60 (112.90-154.57) | 134.87 (115.50-156.53) | 123.18 (105.81-145.59) | 118.44 (101.90-141.58) | 113.31 (89.74-141.76) | 20.50 |
| North Macedonia | 117.77 (101.80-157.77) | 147.74 (125.09-167.90) | 179.09 (130.09-201.27) | 154.18 (122.62-174.48) | 139.14 (118.13-161.77) | 136.62 (114.84-160.77) | 134.06 (96.44-179.050 | 13.83 |
| Republic of Moldova | 209.69 (178.11-226.53) | 205.15 (180.40-243.73) | 175.65 (163.62-210.70) | 201.14 (185.32-219.86) | 207.31 (186.37-223.63) | 195.83 (166.82-211.96) | 164.02 (132.20-201.63) | -21.78 |
| Romania | 272.58 (255.48-290.89) | 302.26 (269.15-320.49) | 316.36 (252.17-337.23) | 328.44 (242.72-349.16) | 310.31 (217.53-330.86) | 293.86 (201.17-315.28) | 283.47 (195.57-360.22) | 4.00 |
| Serbia | 235.75 (190.86-273.71) | 264.33 (213.12-300.72) | 260.15 (203.09-284.92) | 256.85 (194.90-279.43) | 226.88 (176.79-247.91) | 211.05 (162.58-233.02) | 201.86 (146.57-264.07) | -14.38 |
| Slovenia | 122.40 (89.99-164.88) | 116.83 (91.15-144.97) | 110.16 (98.22-122.28) | 92.91 (84.11-102.13) | 81.03 (72.59-91.48) | 75.66 (65.45-86.83) | 72.30 (53.06-99.05) | -40.93 |

Supplemental file 12. Disability-adjusted life years due to colon and rectum cancer per 100,000 population in the period 1990-2019, estimates with 95% uncertainty interval per country in the Balkan region, and per cent change 2019 vs 1990

| Country | 1990 | 1995 | 2000 | 2005 | 2010 | 2015 | 2019 | 2019 vs 1990 (%) |
| --- | --- | --- | --- | --- | --- | --- | --- | --- |
| Albania | 135.25 (126.49-144.52) | 123.69 (115.59-132.48) | 144.82 (136.58-153.01) | 188.07 (177.04-198.72) | 213.16 (181.11-248.68) | 268.15 (200.35-339.50) | 302.34 (225.05-397.30) | 123.54 |
| Bosnia and Herzegovina | 315.33 (298.58-333.26) | 400.53 (319.60-499.57) | 461.98 (406.87-514.16) | 617.05 (566.38-670.44) | 700.30 (652.65-754.31) | 795.06 (741.96-843.60) | 856.03 (673.97-1072.27) | 171.47 |
| Bulgaria | 680.07 (645.10-713.62) | 548.04 (521.75-574.95) | 601.19 (568.28-631.15) | 867.65 (823.78-908.94) | 1039.09 (986.32-1086.54) | 1095.50 (1031.93-1156.15) | 1125.41 (897.78-1393.01) | 65.48 |
| Croatia | 639.57 (602.03-680.06) | 707.05 (667.67-744.24) | 752.77 (712.39-795.63) | 891.90 (856.29-923.97) | 1001.65 (959.19-1040.69) | 1091.78 (1035.84-1141-55) | 1044.32 (828.89-1305.46) | 63.28 |
| Greece | 407.97 (388.90-424.35) | 465.01 (444.15-483.42) | 505.98 (483.12-527.33) | 516.89 (491.91-539.35) | 523.94 (495.76-549.62) | 585.21 (548.94-614.86) | 642.32 (589.93-689.17) | 57.44 |
| Montenegro | 327.17 (276.26-373.87) | 347.68 (312.96-381.27) | 396.90 (366.77-428.02) | 472.35 (441.05-507.32) | 513.74 (472.68-557.87) | 560.36 (497.55-623.28) | 590.54 (485.85-707.08) | 80.50 |
| North Macedonia | 340.99 (312.06-367.16) | 407.95 (385.86-431.85) | 516.38 (488.60-544.44) | 584.19 (553.59-616.61) | 652.12 (617.18-686.85) | 693.62 (647.53-742.13) | 729.09 (572.89-916.37) | 113.82 |
| Republic of Moldova | 486.50 (467.34-508.09) | 524.15 (502.38-552.91) | 407.92 (392.29-423.04) | 544.83 (522.57-571.11) | 700.52 (672.18-731.04) | 751.58 (720.25-784.53) | 676.68 (588.06-770.54) | 39.09 |
| Romania | 390.83 (376.82-404.57) | 516.50 (498.12-535.94) | 493.80 (472.93-512.26) | 590.92 (571.12-611.32) | 709.53 (682.93-734.57) | 786.21 (752.11-817.00) | 848.20 (695.47-1022.47) | 117.03 |
| Serbia | 596.25 (490.41-668.83) | 659.93 (591.22-714.00) | 727.33 (699.83-758.02) | 862.43 (830.51-896.49) | 921.55 (883.87-961.25) | 955.84 (909.66-1002.86) | 967.17 (771.59-1204.91) | 62.21 |
| Slovenia | 581.89 (448.85-752.48) | 665.47 (545.38-804.30) | 752.81 (715.86-787.67) | 796.26 (755.89-832.78) | 833.77 (784.23-878.33) | 778.51 (717.41-833.66) | 810.36 (630.96-1047.96) | 39.26 |
